# Data-Driven Analysis of Fluorescence Lifetime Imaging Experiments: Unraveling the Signal/Stress Relationship of Polluted Microalgae Cells with Machine Learning

**DOI:** 10.1021/acsomega.5c04304

**Published:** 2025-06-06

**Authors:** Erwan Privat, Ilaria Fortunati, Camilla Ferrante, Sergio Rampino, Antonino Polimeno

**Affiliations:** Dipartimento di Scienze Chimiche, Università degli Studi di Padova, Via Marzolo 1, Padova 35131, Italia

## Abstract

Chlorophyll *a* fluorescence decay profiles of biological cells may be used as indicators of the ability of a plant to tolerate environmental stress and the extent of the associated damage to its photosynthetic apparatus. However, the interpretation of data remains often complex and sometimes controversial. Based on previously recorded experimental data from fluorescence lifetime imaging microscopy (FLIM) on the freshwater microalga Coccomyxa cimbrica exposed to the Cu­(II) toxic agent, in this work, we set out to investigate the relationship between FLIM measurements and cell stress conditions based on a data-driven approach. In particular, we analyze the changes induced by Cu­(II) in the photosynthetic cycle of the microalga by monitoring the decay profiles of single cells exposed to different concentrations of Cu­(II) (0, 30, 100, 300, 500, and 700 μg mL^–1^) as a function of time (0, 24, 48, 72, and 96 h) and use Machine Learning to train predictive models mapping the signal shape to Cu­(II) dosage (defined here as the product of the concentration of Cu­(II) and the time of exposure to it) and to gain insights into the signal features that are more deeply connected with the cell health status. Results show that a good tabularization of the data can lead to acceptable predictions with several standard models, with random forest and ridge regressors showing the best performances. Feature-importance analysis of the forest model reveals that a few statistical features of the fluorescence signal, in combination with its decay rate, are the most relevant descriptors. A final analysis of the predictive performances of more sophisticated models, including fully connected and convolutional neural networks, confirms that careful feature engineering coupled with simpler ML models can lead to equally good performances in shorter training times.

## Graphical abstract

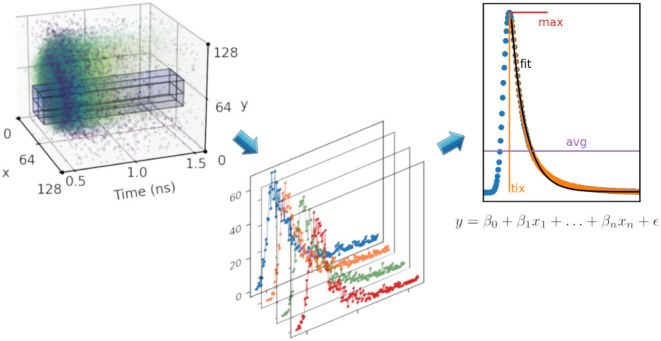

## 1 Introduction

Microalgae are unicellular organisms present in marine and fresh water environments. Besides playing an important role in the production of oxygen through their photosynthetic cycle, these organisms have attracted the attention of scientists due to their capability of producing lipids, and thus biofuels^1^ and their potential versatility in the removal of toxicants in fresh waters, given their high tolerance toward many inorganic and organic toxic agents.^2^

The health status of the photosynthetic apparatus of microalgae can be potentially assessed by chlorophyll *a* fluorescence measurements^3^ and several studies have been devoted to this topic.^4–9^ In particular, the observation of changes in the spontaneous fluorescence can be put in relation with stress conditions of the cells and with nonoptimal functioning of their photosynthetic apparatus. However, the interpretation of data remains often complex and at times controversial.^4^ Among toxic agents, the metal ion Cu­(II) is known for its essentiality for optimal metabolism and is indeed an essential micronutrient with a strong effect on the growth of aquatic microorganisms, including microalgae. However, high doses of Cu­(II) cause adverse effects such as the production of ROS (Reactive Oxygen Species), which in turn affect the metabolic pathways and the photosynthetic efficiency of microalgae.

In a recent work coauthored by some of the present authors^10^ the effect of increasing doses of Cu­(II) on the freshwater microalga Coccomyxa cimbrica (C. cimbrica)^11^ was tracked in time by confocal fluorescence imaging microscopy. In those experiments, the intensity of spontaneous chlorophyll emission from single cells was measured, and the statistical distribution of these single-cell emissions was analyzed. In addition, Fluorescence Lifetime Imaging Microscopy (FLIM) was performed to gain some insight in the change of the decay path of chlorophyll *a* for microalga C. cimbrica when exposed to increasing doses of Cu­(II). Results suggested indeed a relation between the average fluorescence lifetime and both the concentration of Cu­(II) and the time of exposure to it.

In the present article, based on the above-mentioned previously recorded experimental FLIM data, we set out to investigate the relation between FLIM measurements and cell stress conditions based on a data-driven approach. In particular, we analyze the changes induced by Cu­(II) in the photosynthetic cycle of the microalga by monitoring the decay profiles of single cells exposed to increasing concentrations of Cu­(II) (0, 30, 100, 300, 500, and 700 μg mL^–1^) as a function of time (0, 24, 48, 72, and 96 h), and use Machine Learning (ML) to train predictive models mapping the signal shape to Cu­(II) exposure and concentration, and to gain insights into the signal features more deeply connected with the cell health status.

It is worth mentioning here that the use of ML in FLIM applications has been recently explored mainly in relation to generating FLIM images (see Section 2.2) quickly through neural networks rather than by least-squares fitting,^12–16^ The focus of this work is instead rather on the analysis and interpretation of FLIM results. Accordingly, the article is organized as follows. In Section 2, the protocol used to obtain the data analyzed in the present work is briefly summarized, and details on the developed ML software and the related GitHub code and data repository are given. In Section 3, we discuss the predictive power of several selected models and perform an analysis of the features that are more relevant to the predictivity of the models. In Section 4, the main results are summarized and some conclusions are drawn.

## 2 Methods

### 2.1 Experimental Setup

The experimental data analyzed in this article were previously obtained by some of us according to the protocol described in ref. 10. For the reader’s convenience, we briefly summarize it here. Samples of microalga C. cimbrica were grown in Murashige and 1/2 Skoog medium with the addition of sucrose (2% *w*/*w*) and buffered at pH 5.5. All cultures were kept at room temperature under a photoperiod of 16 h of light and 8 h of darkness. Cultures of C. cimbrica contaminated with different concentrations of Cu­(II) in the range from 10 to 700 μg mL^–1^ were prepared using CuCl_2_ from Merck. All experiments were performed on fresh C. cimbrica samples in the exponential growth phase. 10 mL of cell cultures exposed to CuCl_2_ solution of different concentrations were sealed in plastic falcons, which were constantly stirred by an orbital shaker. For the fluorescence experiments, 0.5 mL of sample was collected each time and sealed in homemade glass cells which were afterward discarded.

The spontaneous fluorescence of chlorophyll *a* present in the algae was observed with a laser scanning confocal fluorescence microscope (Olympus Fluoview FV300, Milan, Italy), that allowed recording images of the fluorescence intensity as well as the FLIM maps for single cells. For the fluorescence-intensity experiments discussed in ref. 10 cells were excited by a continuous-wave Argon laser at 488 nm with average power on the sample of 4 μW. The FLIM measurements that will be discussed in this article were done with the same confocal microscope but excited with a frequency doubled Ti-sapphire laser emitting 150 fs long pulses at 410 nm with a repetition rate of 76 MHz. The laser beam was focused into the sample with a 60X water immersion objective, allowing for an average power on the sample of 40 μW. For FLIM maps, a 600 nm long-pass filter was placed in front of a single photon avalanche photodiode (SPAD DPM from MPD, Bolzano, Italy) to record the signal. TCSPC fast electronic from Picoquant, Berlin, Germany, (PicoHarp 300) was used to record the fluorescence decay curves. FLIM maps were recorded for single cells on an area of 128 × 128 pixels with each pixel having dimension of 100 nm. For each of the considered Cu­(II) concentrations (0, 30, 100, 300, 500, and 700 μg mL^–1^) and times of exposure (0, 24, 48, 72, and 96 h), FLIM measurements for 10 different C. cimbric *a* cells are considered, for a total of 6 × 5 × 10 = 300 FLIM experimental outputs.

### 2.2 Data Collection and Curation

The original data set resulting from the FLIM experiments consisted of 700 PicoQuant.ptu files containing raw Time Tagged Time-Resolved (TTTR) fluorescence signals for each cell at different Cu­(II) concentrations and exposure times, resulting in a total of 20.7 GB of data. These 700 experimental outputs included the above-mentioned subset of 300 experiments plus additional test experiments not used in ref. 10, and thus not considered in this work. For each FLIM experiment, photon counts of each pixel as a function of time on an evenly spaced grid from *t* = 0 to *t* ≈ 13 ns were extracted from 699 out of 700 .ptu files (one of the .ptu files was in fact corrupted) using the readPTU_FLIM Python library.^17^

The left panel of Figure 1 schematically shows the content of a single .ptu file, i.e., a color-coded fluorescence decay curve for each illuminated pixel of the microscope grid. In typical FLIM applications, each of these fluorescence decay curves is fitted with a multiexponential function *I*(*t*) = ∑_*i*_ *A*_*i*_ exp­(-*t*/τ_*i*_) where *I*(*t*) is the fluorescence intensity as a function of time, and τ_*i*_ and *A*_*i*_ are the characteristic decay time and the associated amplitude, respectively. Starting from these parameters, different types of images of the cells can be generated, including color-scale maps showing the intensity-weighted average decay time and the amplitudes associated with the characteristic decay times. However, as revealed by a visual inspection of the FLIM images discussed in ref. 10, the specific shape and geometric features of the cell displayed by the generated image will typically depend on contingent aspects related to the actual status of the cell (e.g., orientation, life-cycle stage) at the moment of the experiment and not necessarily related to Cu­(II)-induced cytotoxicity. The inclusion of these features may thus actually hinder the learning process of the relationship between the experimental signal and the stress conditions of the photosynthetic apparatus due to exposure to Cu­(II). Therefore, as already done in ref. 10, we chose to compact the information resulting from a single FLIM experiment for an area of 128 × 128 pixels (left panel) into a single “overall” decay curve (right panel) by summing together, for each time step, the photon counts of all pixels. This resulted in an operational data set containing, for each of the FLIM measurements, the following information: experiment ID serial number (“sample ID” hereinafter), array of the time grid in ns, array of the associated overall photon counts, date of the experiment, time of exposure to Cu­(II) in hours, concentration in μg mL^–1^, cell ID serial number (from 1 to 10, labeling each of the 10 different cells analyzed at a given time of exposure and at a given concentration). The resulting data set (see Figure 2 for a synoptic view showing the first five and the last five of its entries) was saved and stored as a standalone file in JSON format totaling 20.5 MB, and is made available in the GitHub public repository associated with this work (see Section 2.3).

**Figure 1:**
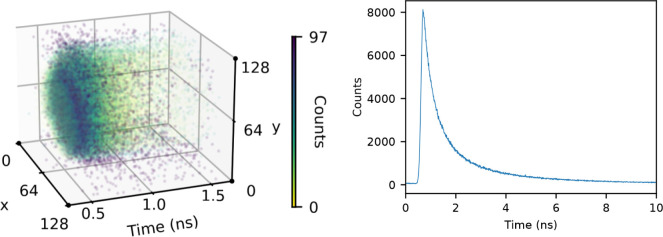
Schematic picture of the results of a FLIM experiment. Left panel: pixel-resolved photon counts as a function of time (note that for better visualization only data for a narrow time frame are shown). Right panel: overall photon count as a function of time up to 10 ns.

**Figure 2:**
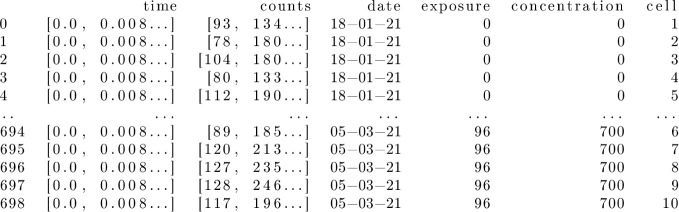
Synoptic view of the flimdf.json data set.

Out of the 699 experiments (also including, as already mentioned, additional test experiments not used in ref. 10) only the relevant subset of 300 experiments (sample IDs from 249 to 548) detailed at the end of Section 2.1 was retained for conducting the analysis presented in this article. For these experiments, the time grid featured a spacing Δ*t* = 0.008 ns and a variable number of points comprised between 1643 and 1649 points. We conformed the data such that all photon-count arrays have the same size by trimming all the arrays down to the first 1643 elements (since, as we shall see, the photon counts exhibit exponential decay, the very end of the tail can be safely discarded with no impact on the statistical features of the signal).

### 2.3 ML Analysis and Software

Our main aim is to build a model capable of predicting the intoxication status of the cells, in terms of concentration of Cu­(II) and exposure to it, from the shape of the overall decay signal. While in principle we could take as features the whole time and counts time series, a more efficient approach is that of collapsing this series to tabular data so as to avoid having to deal with array-like data. We do this by extracting the following statistical features (the actual name in the code is given in parentheses) for each counts array: mean (counts_avg), standard deviation (counts_std), skew (counts_skew), maximum value (counts_max) and location in time of the maximum value (counts_tix). We augment this set of features by adding information deriving from a simple exponential fit as shown in Figure 3 for the decreasing part that is below 95% of the maximum of the signal.

**Figure 3:**
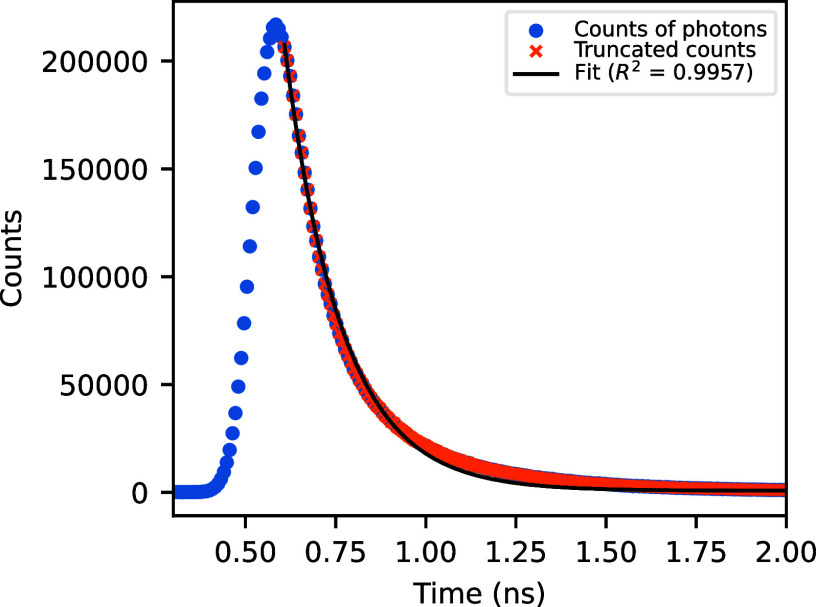
Sample photon counts plot (dots) with exponential decay fit (solid black) of the decreasing part of the signal that is under 95% of the maximum (crosses).

The fitting function is of the form

f(t)=aexp(−bt)+c 1

and we keep only the decay rate *b* (fit_rate) and the constant term *c* (fit_const) since the amplitude information *a* is already encoded in the average or the maximum value of the signal. Additionally, we include interaction features, i.e., the product of each pair of features, so that we can better account for nonlinearities. Apart from purely numerical reasons, it could be argued that the photon count absolute value may depend on the specificity of the cell emissivity and experimental noise, and that the signals should be normalized in some way. We were not able to detect such bias after comparing results before and after the scaling of the counts feature. We discuss in more detail the relative importance of combination of features in Section 3.2.

Analysis and interpretation were conducted using the Python code available at the GitHub repository https://github.com/srampinogroup/flim-ccimbrica based on Python common libraries such as Numpy, Pandas and Matplotlib, and on the ML toolkit scikit-learn^18^ and the Keras^19^ library among the others listed in file requirements.txt in the repository, where also specifics on package versions are reported.

## 3 Results and Discussion

In a preliminary analysis, we explored different candidate standard models that we could envisage as suitable for our problem. After several tests, we decided to focus on four models. They are given as follows with an abbreviation used in this paper and the scikit-learn type used in our code:

- “Lin” LinearRegressor, standard linear regressor,
- “Rid” Ridge, ridge regressor, that is linear regressor with regularization,
- “For” RandomForestRegressor, random forest^20^ regressor,
- “GBR” GradientBoostingRegressor, gradient boosting^21^ regressor.

We anticipate here that the random forest (which is still state-of-the-art for tabular data^22^) and the ridge regressor are found to be the most performant models. We will however show the results for all of the four considered models in order to discuss general trends.

As already mentioned, our final aim is the prediction of at most two target labels (concentration of Cu­(II) and exposure to it) from a selected set of features of the overall decay curve. As the concentration and exposure labels come in discrete sets, our problem can be treated either as a classification task or a regression task. While in a preliminary stage we explored both classification and regression models, we decided to eventually focus on the regression task: it indeed still makes more sense to consider our labels as real values and to keep the numerical relationship between the values, which would be lost in a classification approach. An important reason to choose a regression approach is in fact to keep our model general. A classification approach would indeed restrict the present work to experimental data produced using exactly the same set of fixed values for concentration and exposure times, whereas by allowing full ranges for these parameters, data obtained by extending or replicating experiments using different amounts of Cu­(II) can be both integrated straightforwardly into the training process and be directly taken as input of an already trained model.

We organized our work in three stages. In a first exploratory stage (Section 3.1), in order to reduce the dimensionality of our problem we focused on subsets of the complete data where one of the labels (concentration or exposure time) has a fixed value in order to gain insight into the predictability of the other label. In a second stage (Section 3.2) we moved to the crafting of simple predictive models mapping the signal features to the product of the concentration and the exposure time. This quantity would formally be called the Cu­(II) exposure,^23^ but to avoid confusion between exposure and exposure time we decided to refer to this product as the “dosage”. In a final stage (Section 3.3) we pushed the modeling to a Neural Network (NN) approach to assess to what extent the adoption of more flexible, albeit less interpretable, ML models can improve performance on predictivity.

For all models, cross validation was made on 80% of the data while the remaining 20% was reserved for testing the model on unseen data (i.e., data that have not been used for the training). On those 80%, repeated stratified K-fold cross validation is applied: the set is cut into five folds, that is subsets of that data, of which one is selected as validation set for validation of the model and computation of the regression scores *R* ^2^ and Mean Absolute Error (MAE). This process is repeated ten times. The cross validation scores are the final *R* ^2^ and MAE scores averaged over each run. The test scores are computed on the test dataset set aside in the previous step. Unless specified otherwise, the figures labels show the test score. Cross-validation scores are gathered in Table 1. In order to make the results of this paper reproducible, every computation was done by setting to the arbitrarily chosen seed 1 the “random state” parameter for every relevant call, and by seeding both Numpy and Keras with the same seed. Additional details (e.g., on hyperparameters) for the adopted NNs are given in Section 3.3.

**Table 1:** Scores and Variation across Folds for Repeated Stratified K-Fold Cross-Validation^a^

	*R*^2^ mean	*R*^2^ std.	MAE mean	MAE std.
Concentration fixed at 0 μg mL^–1^:				
Lin	–0.08	0.26	29	3
Rid	0.00	0.41	25	4
For	–0.06	0.13	28	2
GBR	–0.06	0.44	22	6
Concentration fixed at 700 μg mL^–1^:				
Lin	0.48	0.18	18	3
Rid	0.72	0.11	13	2
For	0.45	0.08	19	2
GBR	0.60	0.24	13	4
Exposure time fixed at 0h:				
Lin	0.05	0.28	206	29
Rid	–0.51	1.04	236	52
For	–0.06	0.16	200	25
GBR	–0.34	0.44	225	52
Exposure time fixed at 96 h:				
Lin	0.47	0.26	138	33
Rid	0.63	0.14	123	24
For	0.60	0.16	113	24
GBR	0.39	0.30	128	37
Dosage:				
Lin	0.55	0.08	8247	748
Rid	0.66	0.06	7064	753
For	0.69	0.07	5956	766
GBR	0.65	0.08	6241	885

a Units of MAE are h for fixed concentration, μg mL^–1^ for fixed exposure time, and μg mL^–1^ h for dosage.

### 3.1 Exploratory Analysis with Fixed Concentration or Exposure

The performances (in terms of *R* ^2^) of the four selected models in predicting exposure times for fixed concentrations of 0, 500, and 700 μg mL^–1^ are summarized in the three panels of Figure 4. Analogous plots in Figure 5 show the performances of the same models in predicting concentrations for fixed exposures of 0, 24, and 96 h. Note that in both Figures 4 and 5 (as well as in Figure 8 of Section 3.2) the reported *R* ^2^ score is the average test *R* ^2^ score over the ten different cross-validation runs, while the plotted predicted versus actual values are necessarily those related to only one fold of one of the ten runs, since the train/validation split and hence the samples do change from run to run.

**Figure 4:**
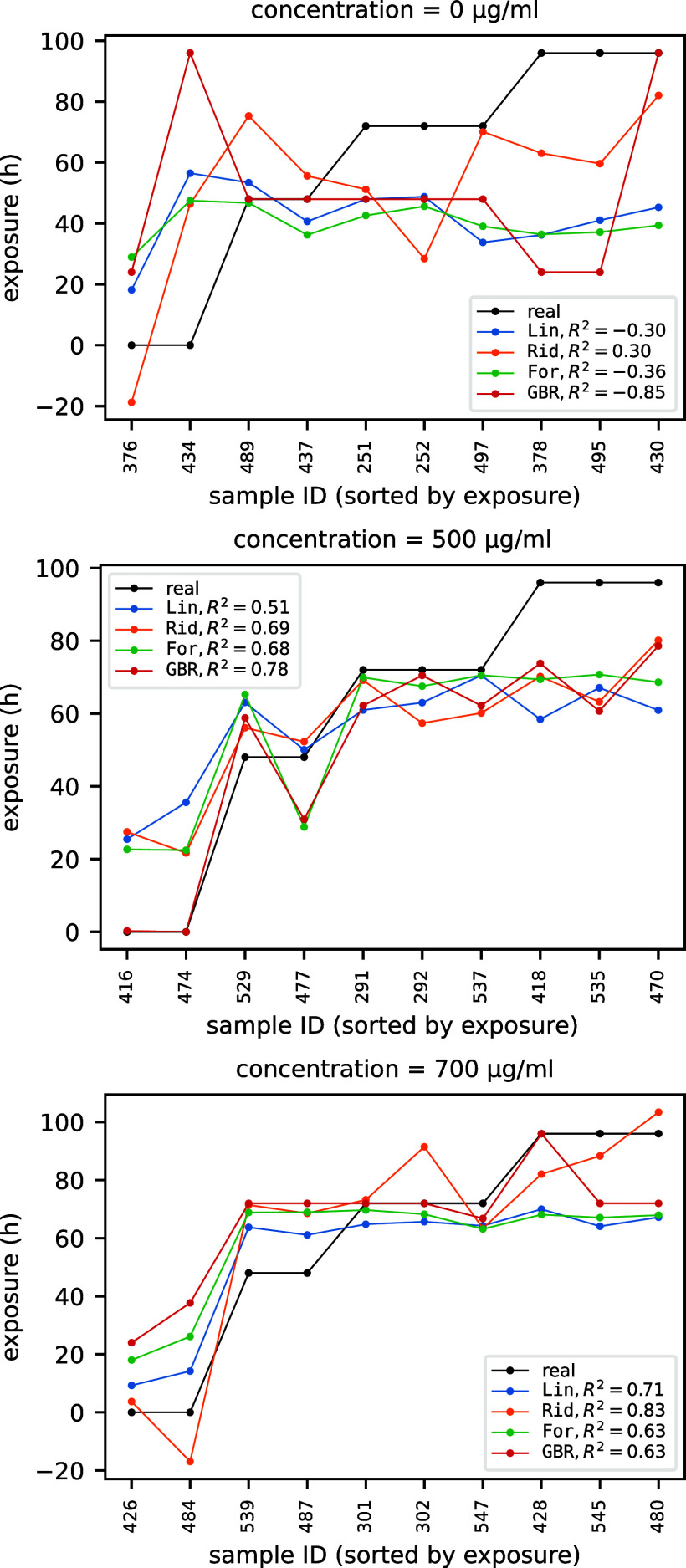
*R* ^2^ score of predicted exposure for fixed concentrations of 0, 500, and 700 μg mL^–1^.

**Figure 5:**
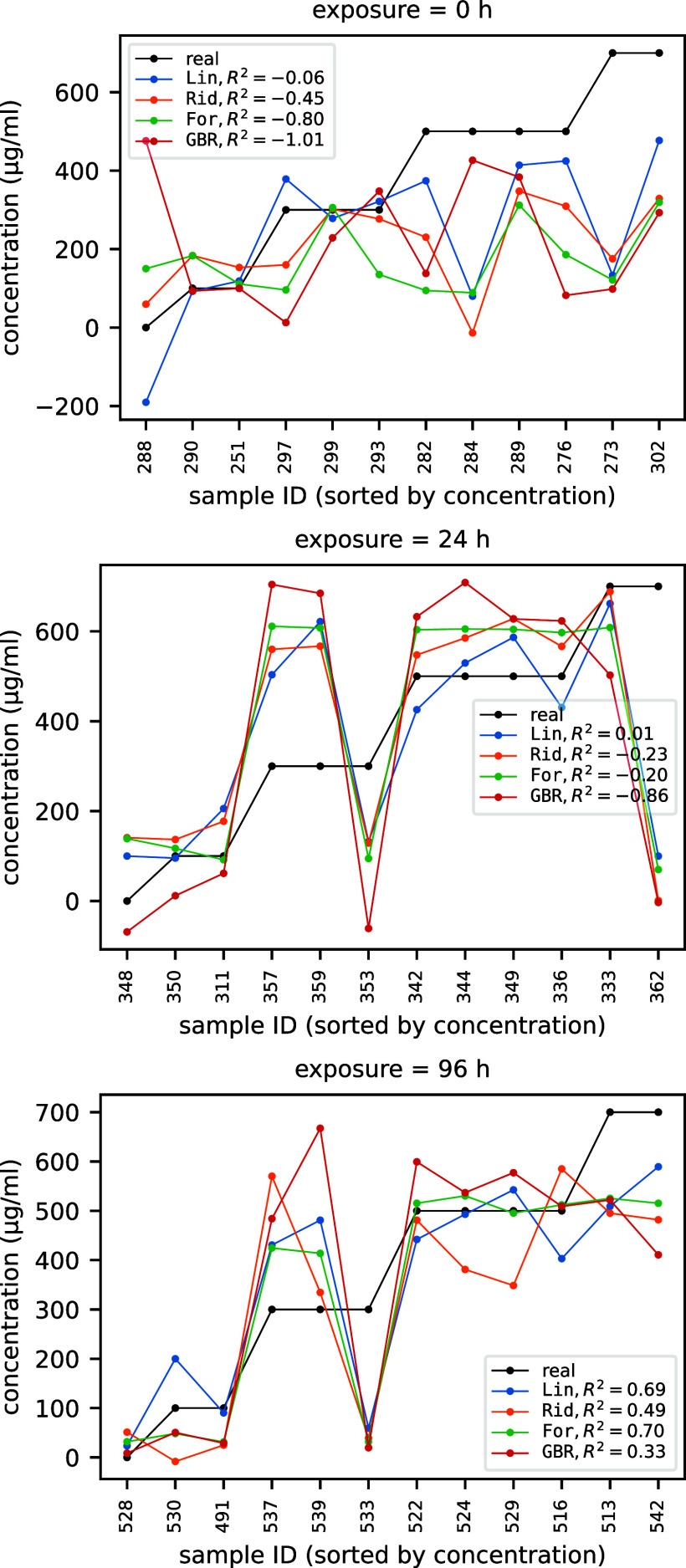
*R* ^2^ score of predicted concentration for fixed exposures of 0, 24, and 96 h.

It should be stressed here that having a single target label for prediction simplifies interpretation and makes easier to identify trends. However, this comes at the cost of dealing with fewer data points, and in cases such as ours where the amount of available data is limited, this approach can be problematic, as evidenced by the relatively low accuracy of predictions. Such analysis however still provides valuable insights into which features may be considered relevant. The two target labels (concentration and exposure) are in fact strongly correlated, and fixing one of them does not significantly alter the relationship with the signal features allowing for a less cluttered interpretation.

When the concentration is zero (top panel of Figure 4), the exposure observable makes little sense because there is nothing to be exposed to. Similarly, when the exposure time is zero (top panel of Figure 5), the impact of whatever concentration of Cu (II) is minimal and any predictive accuracy should be ascribed to experimental bias/inadvertent effects. Moving to nonzero concentrations (central and bottom panel of Figure 4), as a quite general trend the predictivity of the models increases in going from concentration 500 μg mL^–1^ to 700 μg mL^–1^ leading to acceptable *R* ^2^ scores (reaching for instance 0.83 for the ridge regressor). A similar increasing trend is observed for the *R* ^2^ score values obtained at fixed concentrations, though with more marked differences between the models and with an overall lower predictivity which becomes barely acceptable only on for exposure time equal to 96 h.

The above-discussed trends can be better assessed by inspecting Figures 6 and 7, where the *R* ^2^ score and the MAE, respectively, of the four models for fixed concentrations (left panels) and fixed exposure (right panels) are reported as a function of the exposure and concentration values, respectively. In both cases, having a high value of the fixed variable generally improves the predictive power of the model. More in detail, for the prediction of exposure (left panel) at fixed concentration having higher values of concentration leads to increasingly better performance. On the other hand, this trend is much less clear for the prediction of concentration (right panel) at fixed exposure.

**Figure 6:**
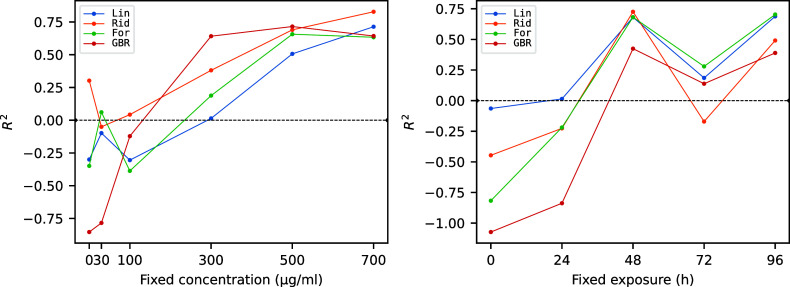
*R* ^2^ score for prediction of exposure for fixed concentration (left) or prediction of concentration for fixed exposure (right) on test data.

**Figure 7:**
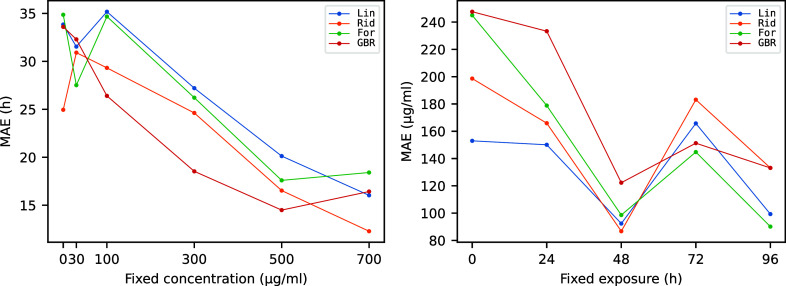
MAE for prediction of exposure for fixed concentration (left) or prediction of concentration for fixed exposure (right) on test data.

Another way to appreciate the increase in accuracy of the models is by looking at the cross-validation scores, which are summarized in Table 1. The variation of the scores across folds (in terms of *R* ^2^ std.) is quite high when the fixed concentration or exposure time is low, indicating (along with the low average scores) that indeed the models fail to find meaningful correlation between features and target. On the contrary, the variation across folds is way lower for high concentration or exposure time, indicating higher prediction stability. The variation becomes even smaller (*R* ^2^ std. comprised between 0.06 and 0.08) for the prediction of the dosage, which will be discussed in the following subsection.

### 3.2 Predictive Models for Dosage

We should note that it is surely the case that we could train and fine-tune a specific model to work better on fixed exposure/fixed concentration, but since the accuracy also depends on the value of the fixed label, it is certainly more interesting to have a global analysis using the same models considered so far but working now on the full set of available data. As a first option, we considered predicting both exposure and concentration with a multioutput regressor, but the strong correlation between the two target labels indicates that it is difficult to discriminate effects coming from each of them. Therefore, we decided instead to look at the product of the two target labels rather than the two labels separately. This allowed to keep the complete set of data in the training of the model, and to predict a biologically interpretable quantity that has the relevant units of a dosage (μg mL^–1^ h). Figure 8 summarizes the performance of the four selected models in predicting the dosage from the signal shape.

**Figure 8:**
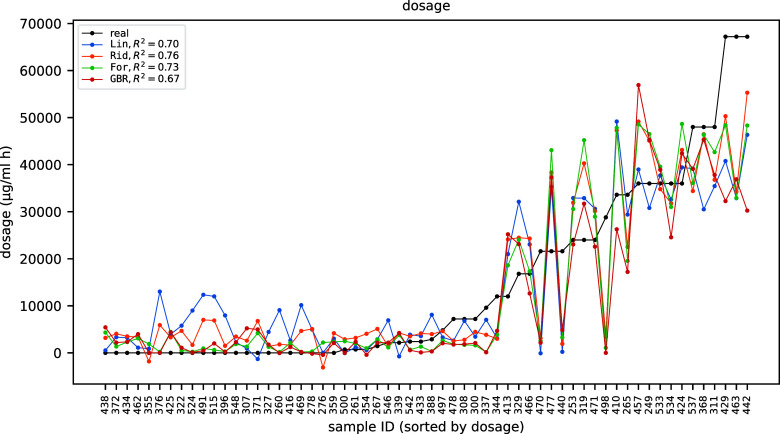
Comparison of real versus predicted dosage.

Within the limitations imposed by the amount of available training data, the models show satisfactory predictive capabilities, with *R* ^2^ score reaching 0.75 for the ridge regressor and 0.73 for the random forest. The accuracy for low values of dosage is also still acceptable because the models have been set to produce only positive prediction when possible (the random forest regression is also naturally constrained to the domain of the training data set). Relative Gini importances of features computed using the built-in implementation of scikit-learn are shown in Figure 9 for a selected forest.

**Figure 9:**
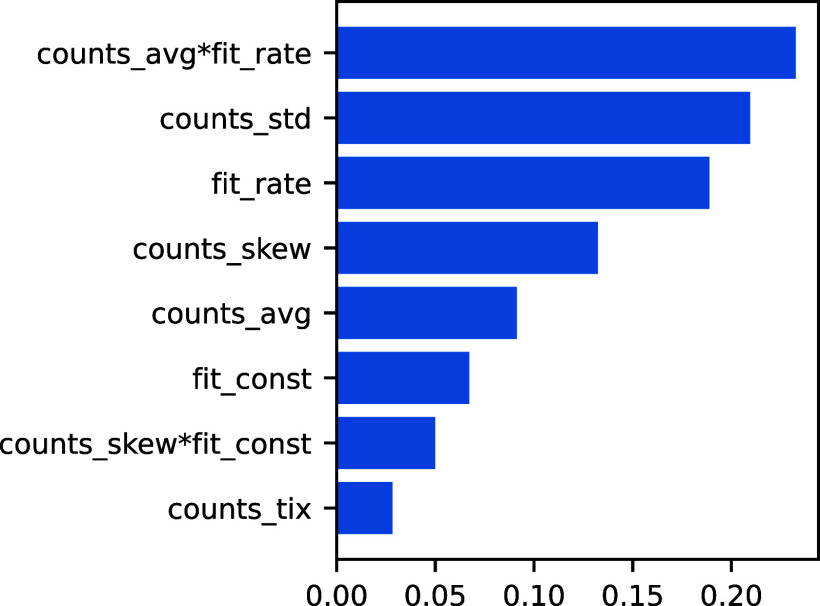
Feature Gini importances of forest regressor for the top eight features. The feature names are the one used in the code, where a star denotes multiplication. The feature counts_tix is the position in time of the maximum.

The average of counts (mainly the scaled absolute value), the decay rate (how fast the curve goes down), the standard deviation (the width of the curve) and the skew (the asymmetry) are the more prominent features for predicting the dosage. These features actually encode almost totally the shape of the curve, meaning that we do not have a lot of redundancy in the features and that the model manages to capture most of the information in the data, further indicating that with more data the predictions could be improved.

### 3.3 Neural Network Approach

We finally explored the adoption of simple and convolutional NNs to investigate to what extent more tunable, though less interpretable, ML models can improve the performances in capturing the signal/dosage relationship.

#### 3.3.1 Fully Connected Neural Network Based on Selected Features

We first adopted a fully connected NN based on the same selection of features used for training the models of Section 3.2, but excluding here the interaction features (product of pair features) as non linearities are already captured by the functional form of the NN by itself.

After a preliminary hyperparameter exploration, we chose to adopt the following NN architecture: the input layers has seven neurons, one for each of the seven features (the maximum and its position in time, average, standard deviation, skew, and fitted parameters decay rate and constant). The hidden layer is comprised of 80 neurons, has a ReLU activation function, and finally the output neuron also has a ReLU activation function since we know that the output should be non-negative. The optimal number of neurons and hidden layers was explored with grid search. Then, based on the above-described optimal NN architecture, we also tested the inclusion of additional hidden layers, with and without dropout. Without dropout the model was seen to overfit, and with dropout it did not perform better than the simpler version that we chose to keep.

As with the previously considered models, we use 5-fold cross validation, and adopt early stopping with big epoch number in the NN training (with an epoch number of 3000, early stopping typically occurred after approximately 1200 epochs). We found that convergence was speeded up by a learning rate of 0.0035 coupled to the AdamW optimizer. Additionally, we performed standardization of the features (such that each feature has mean equal to zero and standard deviation equal to one), which is mandatory in order to have good results with NN models. The performance of the predictive model based on the above-described NN and trained on the selected set of features are summarized in Figure 10.

**Figure 10:**
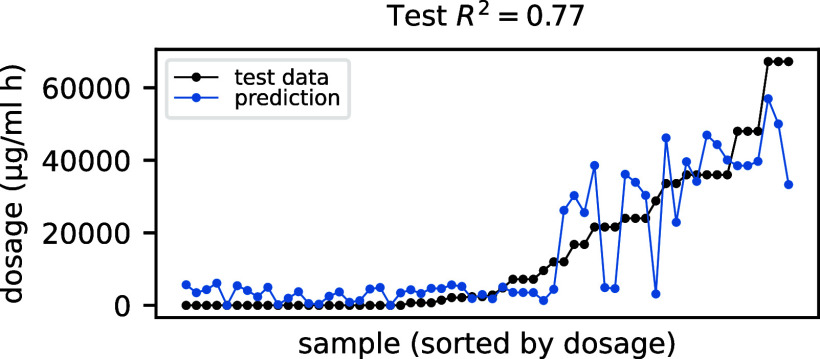
Predicted (blue) vs real (black) dosage as a function of the samples in the test data set (sorted by dosage).

The model shows an *R* ^2^ score of 0.77, which is comparable with that obtained by the linear ridge and random forest regressor in Section 3.2, meaning that, as a result of careful feature engineering, those models were already able to capture any nonlinearity in the signal-dosage relation, and the modeling via the more flexible and less interpretable NNs actually adds only a modest value (some information is still discovered by the NN since the *R* ^2^ score is slightly higher). Incidentally note that, as the simpler models of Section 3.2, the NN model seems to fail in predicting extreme dosages too, indicating that we may be missing some underlying information to properly account for the biological process occurring at high dosages.

For illustrative purposes, we report in Figure 11 the convergence of one cross-validation run for the above-discussed NN, showing that early stopping is triggered at around 1200 epochs. Note that the displayed *R* ^2^ on top of the plot is the one obtained on the test data set averaged over the five considered folds, and that this value is only incidentally equal to that reported in Figure 10 and referring to only one fold. Note also that the fact that the *R* ^2^ curve for the validation data reaches slightly higher values than that of the training data happens for this fold but is not necessarily a general trend. In fact, having a limited amount of data and a rather big (20%) proportion of testing data, some folds of the cross-validation will have “easy” or “hard” validation data corresponding to their training. Other folds (not shown here) exhibit indeed a training score greater than the validation score.

**Figure 11:**
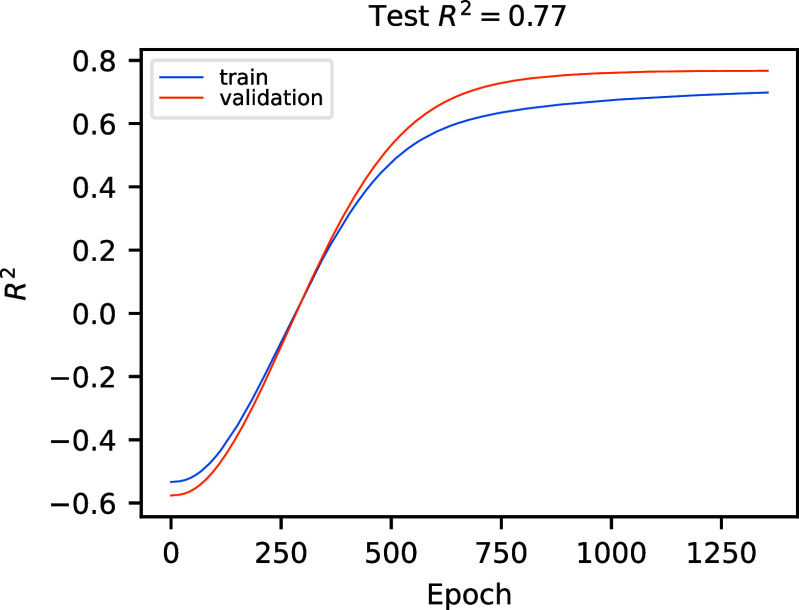
Convergence of one fold of cross-validation of NN.

#### 3.3.2 Convolutional Neural Network and Training Times

We finally explored a brute-force data-driven approach based on a convolutional neural network (CNN). In particular, we trained a one-dimensional CNN on the whole signal (photon counts) time series using one layer of convolution and one dense layer with hyperparameters comparable to the ones used in the previous section for the simple neural network trained on seven selected features. Our analysis and fine-tuning of the CCN revealed that the highest *R* ^2^ scores that could be obtained are of about 0.7, but at the cost of a much higher training time with respect to the previously considered models, further indicating that the feature engineering done for the simpler ML models is sound and that most of the information on the signal/dosage relation is actually encoded in the above-discussed set of selected features.

A summary of the training times, *R* ^2^ scores and MAEs for dosage prediction for all models considered in this work is given in Table 2, for a quantitative assessment of the efficiency/accuracy trade-off. These were evaluated for computations performed on a consumer-grade machine with a dual-core 2.599 GHz CPU, no GPU and 4 GiB of RAM. The simplest models (linear and ridge regressors) are seen to be the fastest to train (in the order of milliseconds) the slowest model is the CNN (>250 s), with the training times for the remaining models falling in the range 20–50 s (note that the training times given for the NNs are those resulting from early stopping, while training the models without early stopping would require approximately ten times more time and lead to almost the same predictive power).

**Table 2:** Training Times for the Considered Models for Dosage Prediction with Associated Test Scores

Model	Lin	Rid	For	GBR	NN	CNN
Time (s)	0.002	0.002	1.147	0.650	23	256
*R* ^2^	0.70	0.76	0.73	0.67	0.77	0.71
MAE (μg mL^–1^ h)	7436	6871	6370	6702	6529	6998

## 4 Conclusions

In this article, we investigate the relation between FLIM measurements and cell stress conditions with a data-driven approach based on a data set of 300 previously obtained fluorescence decay profiles of cells of microalga C. cimbrica exposed to different concentrations of Cu­(II) and recorded at different times of exposure. For a selection of four standard models (linear regressor, ridge regressor, random forest regressor, and gradient boosting regressor), we first focus on predictive models for the concentration of Cu­(II) at fixed exposure time and for the exposure time at fixed concentration of Cu­(II) using relevant subsets of the original data. Then we move to the training and assessment of predictive models for the Cu­(II) dosage, formulated as the product of Cu­(II) concentration and exposure time, using the whole data set.

Results show that a good tabularization of the data can lead to good predictions, in particular with random forest and ridge regressors. The predictivity of exposure time at fixed concentration significantly improves for increasingly higher values of the fixed concentration. The same is true to some extent for the predictivity of the concentration at fixed exposure times: higher fixed exposure times lead to better predictions on the concentrations, but the trend is here less clear. Using the product of exposure time and Cu­(II) concentration (interpreted as the dosage) as the target label leads to satisfactory predictions (*R* ^2^ score of 0.76 for the ridge regressor and 0.73 for the random forest regressor) in relation to the limited amount of available training data. Feature-importance analysis of the forest reveals that a few statistical features of the signal, namely the average photon counts, the standard deviation, and the asymmetry, in combination with a decay rate parameter obtained through a simple exponential fitting are the more prominent features for predicting the dosage.

A final assessment of the performances of more flexible albeit less interpretable models such as NNs confirms that careful feature engineering coupled to simpler models can already saturate the predictive capability extractable from the available training data. Simpler models are in fact seen to lead to as good performances as the NNs due to the fact that the devised set of selected features used for training the formers manages to capture most of the information on the feature-label relationship, making this use case of machine learning satisfactorily sound within the intrinsic limitations imposed by a finite set of available training data.

## Data Availability

The data and code used for the work reported in this article are available at the following GitHub repository: https://github.com/srampinogroup/flim-ccimbrica.
